# Cytotoxic activity of bromodomain inhibitor NVS-CECR2-1 on human cancer cells

**DOI:** 10.1038/s41598-020-73500-7

**Published:** 2020-10-01

**Authors:** Seul Gi Park, Daye Lee, Hye-Ran Seo, Shin-Ai Lee, Jongbum Kwon

**Affiliations:** 1 Department of Life Science, The Research Center for Cellular Homeostasis, Ewha Womans University, 52 Ewhayeodae-gil, Seodaemun-gu, Seoul, 03760 Korea; 2 Present Address: Laboratory of Genitourinary Cancer Pathogenesis, Center for Cancer Research, National Cancer Institute, Bethesda, MD 20892-4263 USA

**Keywords:** Apoptosis, Chemotherapy, Chromatin remodelling, Colon cancer

## Abstract

Bromodomain (BRD), a protein module that recognizes acetylated lysine residues on histones and other proteins, has recently emerged as a promising therapeutic target for human diseases such as cancer. While most of the studies have been focused on inhibitors against BRDs of the bromo- and extra-terminal domain (BET) family proteins, non-BET family BRD inhibitors remain largely unexplored. Here, we investigated a potential anticancer activity of the recently developed non-BET family BRD inhibitor NVS-CECR2-1 that targets the cat eye syndrome chromosome region, candidate 2 (CECR2). We show that NVS-CECR2-1 inhibits chromatin binding of CECR2 BRD and displaces CECR2 from chromatin within cells. NVS-CECR2-1 exhibits cytotoxic activity against various human cancer cells, killing SW48 colon cancer cells in particular with a submicromolar half maximum inhibition value mainly by inducing apoptosis. The sensitivity of the cancer cells to NVS-CECR2-1 is reduced by CECR2 depletion, suggesting that NVS-CECR2-1 exerts its activity by targeting CECR2. Interestingly, our data show that NVS-CECR2-1 also kills cancer cells by CECR2-independent mechanism. This study reports for the first time the cancer cell cytotoxic activity for NVS-CECR2-1 and provides a possibility of this BRD inhibitor to be developed as an anticancer therapeutic agent.

## Introduction

Bromodomain (BRD) is a protein–protein interaction module that consists of about 110 amino acid residues and recognizes acetylated lysine residues on histone tails and other proteins. The human genome encodes 61 BRDs present in 46 distinct proteins, many of which are chromatin regulators, such as chromatin remodeler and histone modifier, that function in a wide array of biological processes. Despite of their diversity of sequences, all BRDs share a conserved fold comprising left-handed four helix bundles linked by the ZA and BC loops, which forms a central deep and narrow hydrophobic cavity for the docking site of acetyl-lysine moiety. The sequence of the ZA and BC loops are highly variable in length and amino acid composition and this structural feature enables different BRDs to specifically bind distinct acetyl-lysine sites in histones and non-histone proteins^1–4^.

Recent studies proved that small compound inhibitors specific for BRDs can be developed as therapeutic agents in treatment of various human diseases, including cancer^5–8^. As the first examples, JQ1 and I-BET were developed to target the bromo- and extra-terminal domain (BET) family comprising BRD2, BRD3, BRD4 and BRDT, and their antitumor activities have been well documented along with some promising results of clinical trials for certain cancer types^9–11^. Subsequently, a number of additional inhibitors for the BET family proteins have been developed and many of these inhibitors are also currently in clinical trials for diverse cancer types^2,12^. The success in evolving potent and highly selective inhibitors for the BET family of BRDs and their entry into clinical trials has stimulated intensive research activities to develop BRD inhibitors targeting non-BET family proteins^7,13,14^. While most of research efforts have been focused on BET family BRD inhibitors, the cellular activity and potential therapeutic applicability of the non-BET BRD inhibitors remains largely unexplored.

One of the recently developed non-BET BRD inhibitors is NVS-CECR2-1, which targets cat eye syndrome chromosome region, candidate 2 (CECR2). CECR2 contains a single BRD and the gene encoding CECR2 is located in the chromosome 22q11 region that is duplicated in the human disorder cat eye syndrome^15–17^. Studies have shown that CECR2 is involved in neurulation and chromatin remodeling as well as DNA damage response^15,18^. NVS-CECR2-1 was developed by the structural genomics consortium (SGC) in collaboration with Novartis and shown in vitro to bind CECR2 BRD with high affinity (IC_50_ = 47 nM, *K*_*D*_ = 80 nM) and selectivity over other 48 BRD targets (https://www.thesgc.org/chemical-probes/NVS-1). Neither the activity of NVS-CECR2-1 against its target within cells nor its therapeutic potential in treatment of human diseases, such as cancer, was reported. Here, we demonstrated that NVS-CECR2-1 inhibits chromatin binding of CECR2 BRD and displaces CECR2 from chromatin within cells. We found that NVS-CECR2-1 has a strong cytotoxic activity on various human cancer cells, killing SW48 colon cancer cells in particular with a submicromolar value of half maximum inhibitory concentration (IC_50_) by increasing apoptosis. Our data showed that the cytotoxic activity of NVS-CECR2-1 on cancer cells is exerted by targeting CECR2 as well as via CECR2-independent mechanism. We also performed analysis of CECR2 gene expression in colorectal cancer tissues using several online databases and discussed the significance of the results.

## Results

### NVS-CECR2-1 inhibits chromatin binding of CECR2 BRD within cells

**Figure 1:**
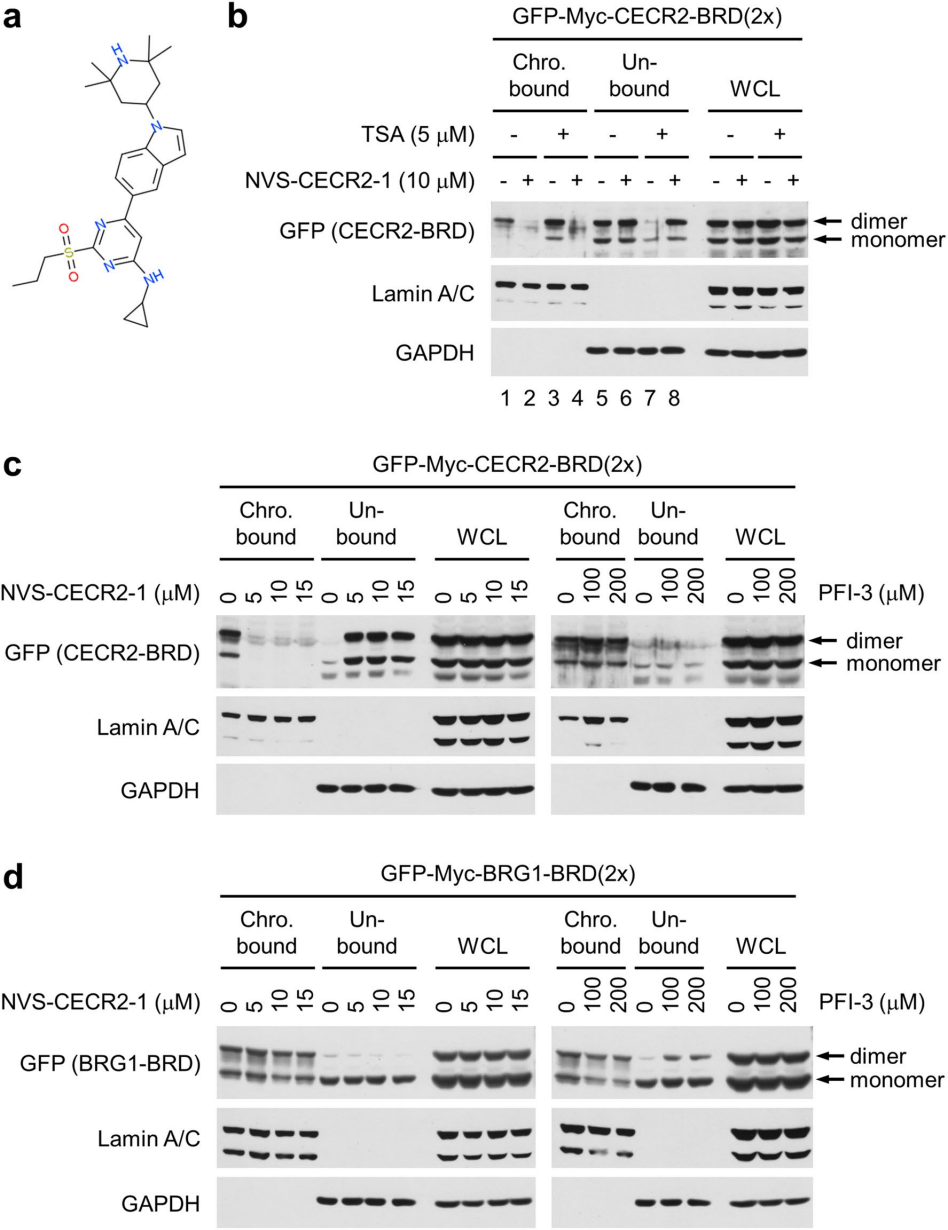
NVS-CECR2-1 inhibits chromatin binding of CECR2 BRD within cells. (**a**) Structure of NVS-CECR2-1. (**b**) SW48 cells were transfected with the vector expressing a dimeric form of CECR2 BRD tagged with GFP and Myc, and treated with NVS-CECR2-1 for 2 h with or without 30-min pretreatment with TSA (5 μM). Cells were harvested and subjected to chromatin fractionation followed by immunoblotting as indicated. Lamin A/C was used as a marker for the chromatin-bound fraction and GAPDH for the soluble unbound fraction. The monomeric forms of CECR2 BRD were produced by degradation of dimeric forms. A representative of three independent experiments showing similar results was shown. (**c**) After transfection with the expression vector as in (**b**) and TSA pretreatment, SW48 cells were treated with increasing concentrations of NVS-CECR2-1 (left) or PFI-3 (right) for 2 h, and subjected to chromatin fractionation as in (**b**). A representative of two independent experiments showing similar results was shown. (**d**) After transfection with the vector expressing a dimeric form of BRG1 BRD tagged with GFP and Myc, SW48 cells were pretreated with TSA and treated with increasing concentrations of NVS-CECR2-1 (left) or PFI-3 (right) for 2 h, and then subjected to chromatin fractionation as in (**b**). A representative of two independent experiments showing similar results was shown. In all drug treatment experiments, the lanes of (−) or 0 μM have DMSO as vehicle.

First, we determined whether NVS-CECR2-1 (Fig. 1a) inhibits chromatin binding of CECR2 BRD. After transfection with the plasmid vector expressing a dimeric form of CECR2 BRD, which binds chromatin with higher affinity than a monomeric form^18^, cells were treated with 10 μM of NVS-CECR2-1 and cell lysates were fractionated into insoluble chromatin-bound and soluble chromatin-unbound fractions. Immunoblot analysis showed that CECR2 BRD roughly equally distributed into the both fractions and this distribution largely shifted to the chromatin-unbound fractions with the CECR2-BRD band in the chromatin-bound fraction barely detected (Fig. 1b, lanes 1, 2, 5 and 6), indicating that NVS-CECR2-1 inhibited the chromatin binding of CECR2 BRD. In parallel experiments, pre-treatment of tricostatin A (TSA) histone deacetylase inhibitor rendered most of CECR2 BRD bound to chromatin, which was nearly completely abolished by NVS-CECR2-1 treatment (Fig. 1b, lanes 3, 4, 7 and 8), further confirming the inhibitory activity of NVS-CECR2-1 against CECR2 BRD. As control, we performed the similar chromatin fractionation experiments for PFI-3, the BRD inhibitor specific for the BRG1 ATPase of SWI/SNF chromatin remodeling complex^19^. Whereas NVS-CECR2-1 effectively inhibited the chromatin binding of CECR2 BRD at the concentrations ranging from 5 to 15 μM, PFI-3 had no activity against CECR2 BRD even at 100 μM or a higher concentration (Fig. 1c). Conversely, PFI-3 inhibited the chromatin binding of a dimeric form of BRG1 BRD, which also has higher chromatin affinity than a monomeric form^20^, whereas NVS-CECR2-1 had little effect (Fig. 1d). These results showed that NVS-CECR2-1 has a potent activity against CECR2 BRD within cells.

### NVS-CECR2-1 dissociates endogenous CECR2 from chromatin

**Figure 2:**
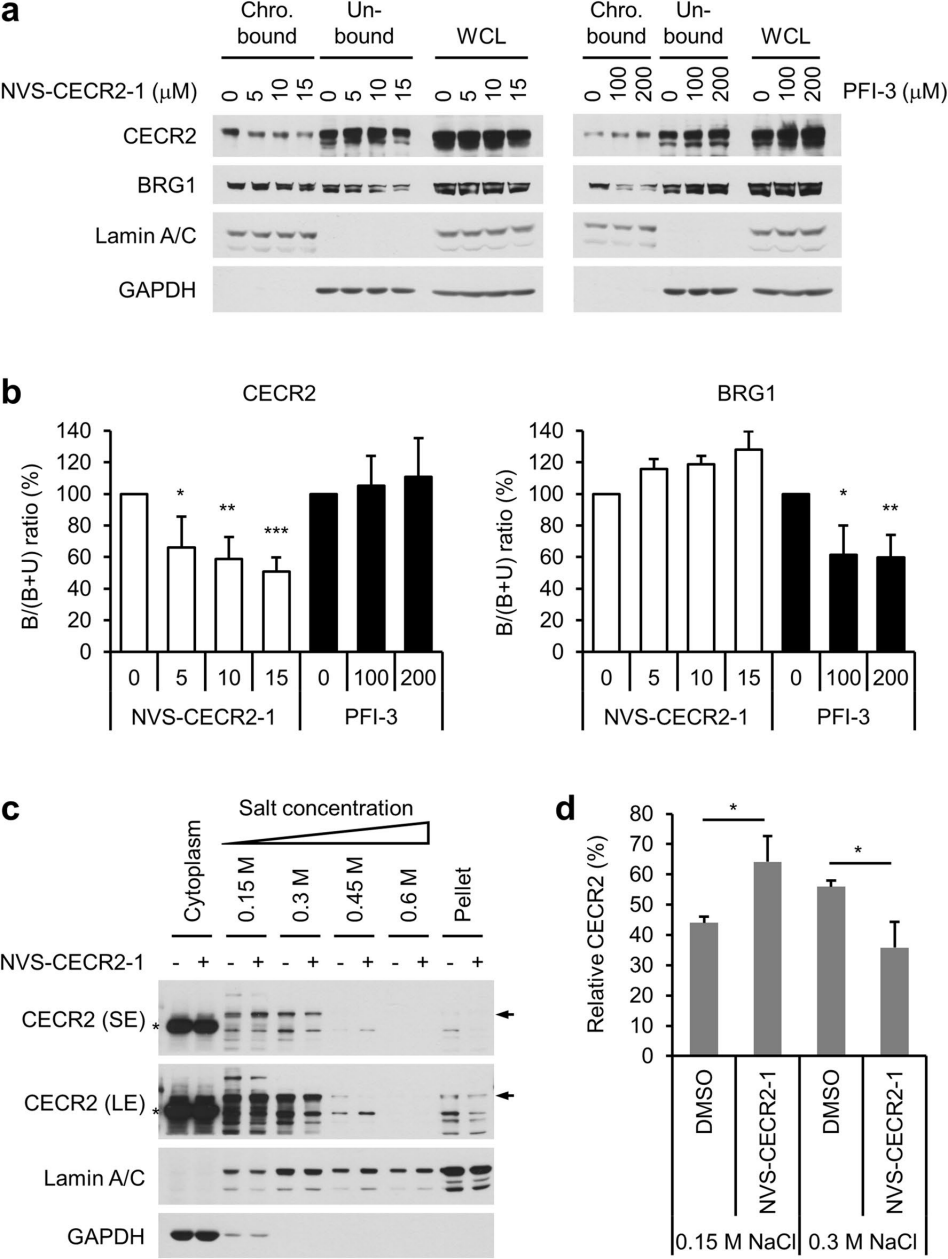
NVS-CECR2-1 displaces endogenous CECR2 proteins from chromatin. (**a**) SW48 cells were treated with increasing concentrations of NVS-CECR2-1 (left) or PFI-3 (right) for 2 h, and subjected to chromatin fractionation as in (Fig. 1b). WCL, whole cell lysate. (**b**) The CECR2 and BRG1 bands of the chromatin-bound (B) and unbound (U) fractions were quantitated by densitometer and normalized to the Lamin and GAPDH bands, respectively. The B/(B + U) ratios of the CECR2 and the BRG1 bands for each experimental condition were calculated and their relative values were graphed. (**c**) SW48 cells were treated with DMSO (vehicle) or 5-μM NVS-CECR2-1 for 2 h, and subjected to salt gradient chromatin fractionation. A representative of four similar results is shown (see Fig. S1). Arrow, CECR2 band; asterisk, nonspecific band. (**d**) The CECR2 bands of 0.15- and 0.3-M salts extractions on the gels in (c) were quantitated with normalization to lamin, and their relative densities were calculated and plotted as graph. *n* = 3; error bars, mean ± s.d. *, *p* < 0.05; **, *p* < 0.01; ***, *p* < 0.001.

Next, we determined whether NVS-CECR2-1 can dissociate endogenous CECR2 proteins from chromatin by chromatin fractionation as described before. The cells were treated with NVS-CECR2-1 at 5, 10 or 15 μM, or with PFI-3 as control at 100 or 200 μM. The results showed that NVS-CECR2-1 dissociated CECR2 from chromatin in a dose-dependent manner without affecting BRG1, and conversely, PFI-3 dissociated BRG1 from chromatin with no effect on CECR2 (Fig. 2a,b). To confirm these results, we conducted salt gradient chromatin fractionation. Most of the cellular CECR2 proteins were extracted by 0.15- and 0.3-M NaCl, and NVS-CECR2-1 treatment increased the CECR2 extraction at 0.15-M NaCl with its concomitant decrease at 0.3-M NaCl (Fig. 2c,d and Fig. S1a–c), indicating that NVS-CECR2-1 dissociated CECR2 from chromatin. As expected from the fact that CECR2 is a chromatin remodeler and thus a nuclear protein, CECR2 was barely detected in the cytoplasmic fractions in salt gradient chromatin fractionation (Fig. 2c,d and Fig. S1a–c) and stained exclusively within the nucleus in immunofluorescence microscopy (Fig. S2a,b). Essentially no CECR2 was found in the soluble nuclear fractions (Fig. S1a,c), indicating that CECR2 exists bound to chromatin in the nucleus. The CECR2 staining pattern remained unchanged after NVS-CECR2-1 treatment in immunofluorescence microscopy (Fig. S2b). These results therefore demonstrated the activity of NVS-CECR2-1 to prevent its target protein CECR2 from binding chromatin within cells.

### NVS-CECR2-1 exhibits cytotoxic activity on various human cancer cells

**Figure 3:**
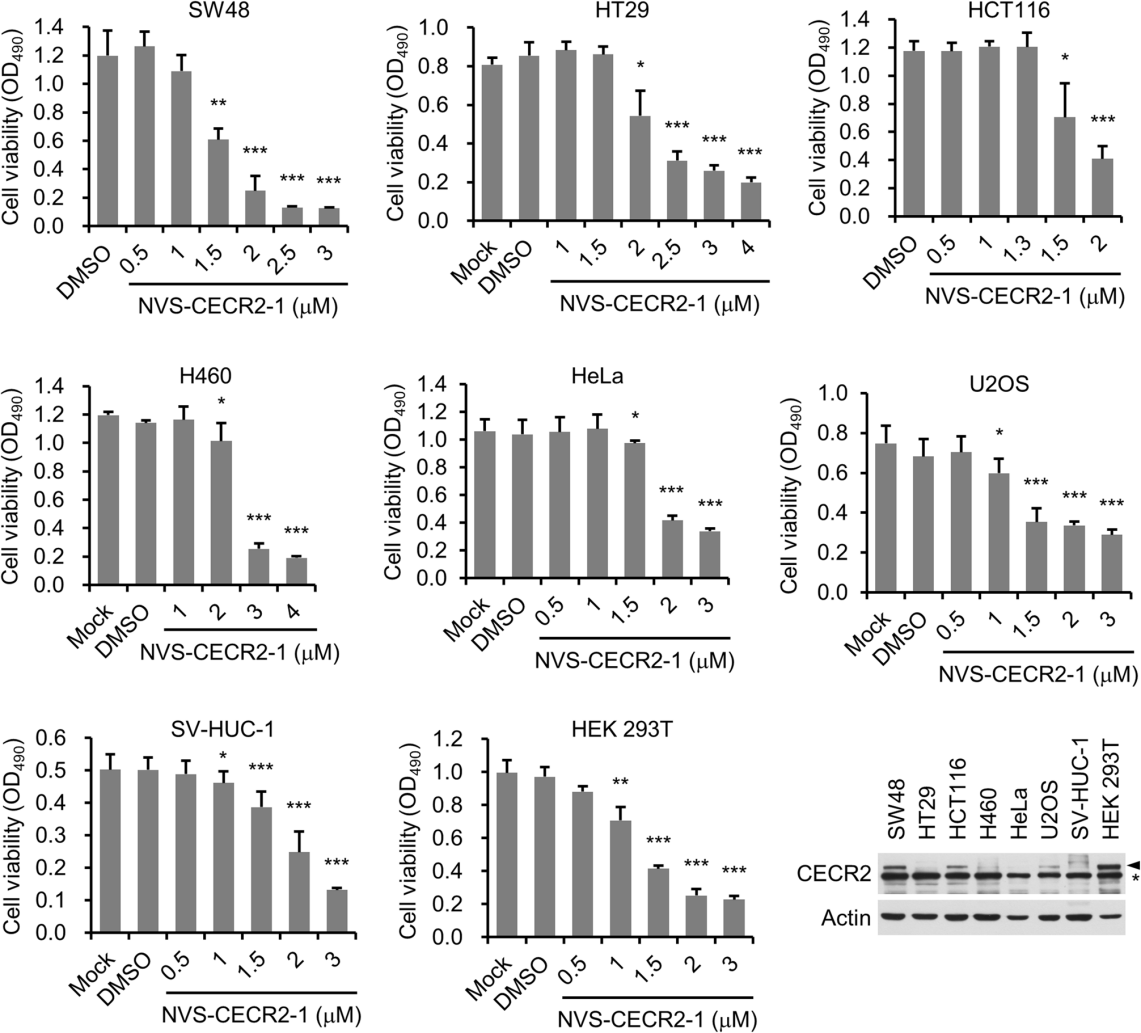
Cytotoxic activity of NVS-CECR2-1 on various human cancer cells. Cells were treated with increasing concentrations of NVS-CECR2-1 for 72 h and their viability was determined by MTS assay. Cell viability was quantitated by measuring the absorbance at 490 nm and depicted as a graph. *n* = 3 (each performed in triplicate); error bars, mean ± s.d. *, *p* < 0.05; **, *p* < 0.01; ***, *p* < 0.001. The last panel shows the results of immunoblot for CECR2 expression of the cancer cell lines described above. Arrowhead, CECR2 band; asterisk, nonspecific band.

Having verified the in vivo activity of NVS-CECR2-1, we investigated the possibility that it kills cancer cells. For this, we selected a set of human cancer cell lines that represent various tissue origins, such as colon (SW48, HT29 and HCT116), lung (H460), uroepithelium (SV-HUC-1), cervix (HeLa) and bone (U2OS). The cells were treated with increasing concentrations of NVS-CECR2-1 and the cell viability was determined after three days by MTS assay. Interestingly, the treatment of NVS-CECR2-1 decreased the viability of all cancer cells analyzed in a dose dependent manner (Fig. 3). NVS-CECR2-1 treatment also showed a dose-dependent cytotoxicity on human embryonic kidney (HEK) 293 T cells, an immortalized cell line originated from non-cancer tissue (Fig. 3). Notably, NVS-CECR2-1 exhibited cytotoxic activity on all the cell types tested at the pharmacologically significantly concentrations, seemingly with a few micromolar concentrations killing more than half of the cell population (Fig. 3). Immunoblot analysis showed that the levels of CECR2 expression varied among the tested cell lines, with some displaying very little (HT29, H460, HeLa and SV-HUC-1) and the others exhibiting different degree of CECR2 expression (SW48, HCT116, U2OS and HEK 293 T) (Fig. 3, last panel). Therefore, the cytotoxic activity of NVS-CECR2-1 on cancer cells is not necessarily dependent on CECR2 expression. At this moment, owing to the limited accuracy of the short-term viability assay, it was difficult to predict any correlation between the cytotoxic activity of NVS-CECR2-1 and CECR2 expression levels of cancer cells.

### NVS-CECR2-1 kills SW48 cells with a submicromolar IC_50_ value by increasing apoptosis

**Figure 4:**
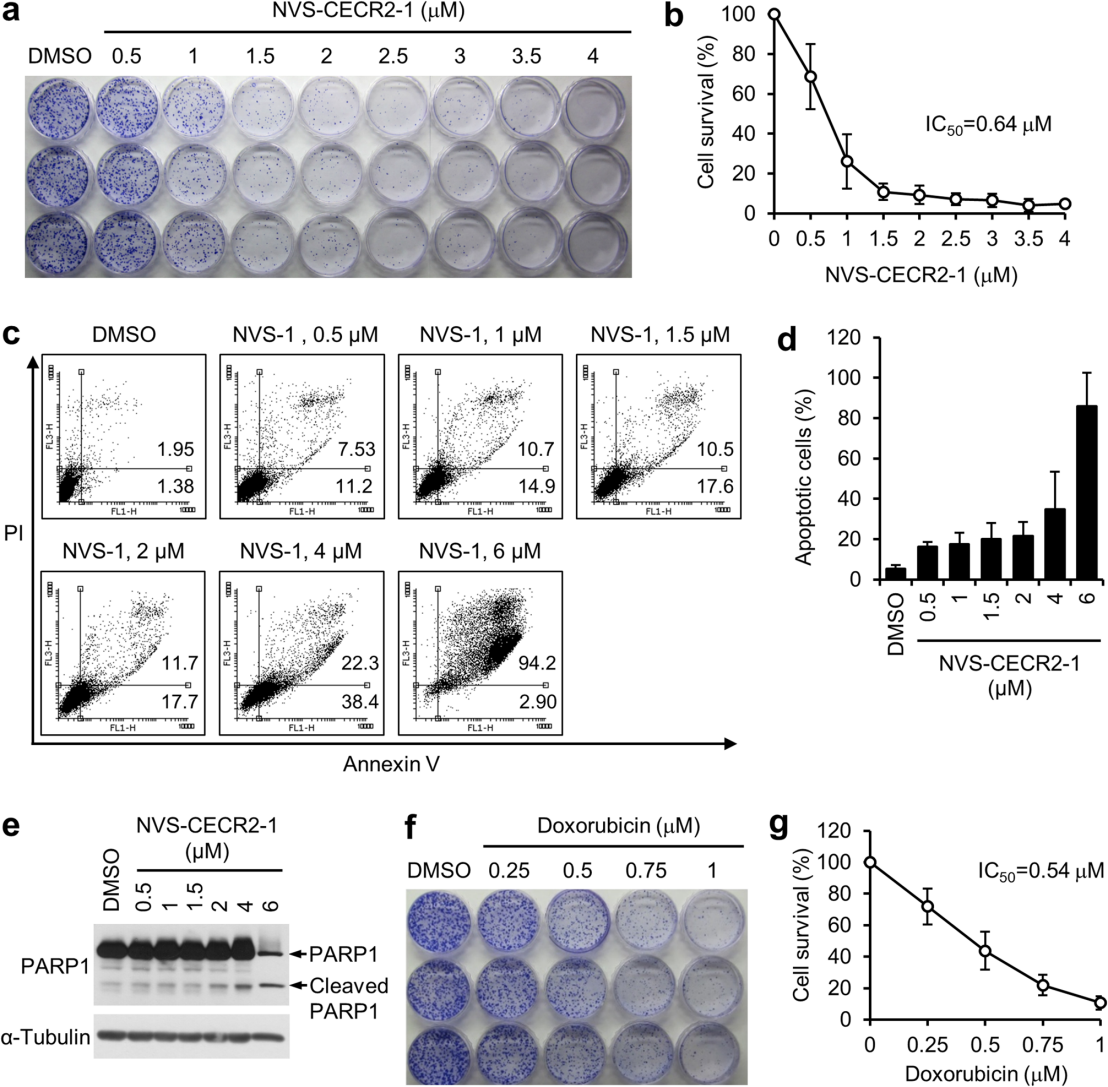
NVS-CECR2-1 kills SW48 cells via apoptosis with a submicromolar IC_50_ value. (**a**) SW48 cells were treated with increasing concentrations of NVS-CECR2-1 and the clonogenic ability was determined by colony formation assay after 10 days. A representative image was shown. (**b**) Results of the colony formation assay were depicted as a graph. The IC_50_ value was shown. *n* = 3 (each performed in triplicate); error bars, mean ± s.d. (**c**) SW48 cells were treated with increasing concentrations of NVS-CECR2-1 for 72 h and subjected to annexin V/PI staining. A representative image was shown along with percentages of early (upper right) and late apoptotic cells (lower right). (**d**) Results of the apoptosis assay by Annexin-V/PI staining were depicted as a graph (sum of early and late apoptosis). *n* = 3; error bars, mean ± s.d. (**e**) Results of the apoptosis assay by PARP1 cleavage. The cells treated as in (**c**) were subjected to immunoblot analysis as indicated. A representative of three experiments showing similar results was shown. (**f**) (**g**) Results of the similar experiments as (**a**) and (**b**) to determine the IC_50_ value of doxorubicin for SW48 cells. *n* = 3; error bars, mean ± s.d.

Since the short-term viability study indicated that NVS-CECR2-1 was most toxic to SW48 cells among the eight cell lines tested, we pursued further investigations for this cell line. First, we determined the effect of NVS-CECR2-1 on the long-term viability of SW480 cells. The cells were treated with increasing concentrations of NVS-CECR2-1 up to 4 μM and their clonogenic ability was evaluated after ten days by colony formation assay. NVS-CECR2-1 inhibited the clonogenic ability of SW48 cells in a dose dependent manner and its IC_50_ value was estimated to be 0.64 μM (Fig. 4a,b). Next, we determined the mechanisms by which NVS-CECR2-1 kills SW48 cells. The cells were treated with increasing concentrations of NVS-CECR2-1 up to 6 μM and analyzed for apoptosis and necrosis after 72 h by Annexin-V assay. NVS-CECR2-1 treatment increased apoptosis in a dose-dependent manner, with more than 80% cells undergoing apoptosis at 6 μM, and had virtually no effect on necrosis (Fig. 4c,d). Increase of apoptosis by NVS-CECR2-1 was confirmed by PARP1 cleavage (Fig. 4e). Therefore, NVS-CECR2-1 kills SW48 cells mostly by inducing apoptosis. For the purpose of comparison, we analyzed the toxicity of doxorubicin on SW48 cells and observed that this well-known potent anticancer drug exhibited the IC_50_ value of 0.54 μM (Fig. 4f,g). These results suggest that, given its IC_50_ value compatible to doxorubicin, NVS-CECR2-1 can be a potentially good candidate of anticancer drug for colon cancer treatment.

### NVS-CECR2-1 kills SW48 cells by targeting CECR2

**Figure 5:**
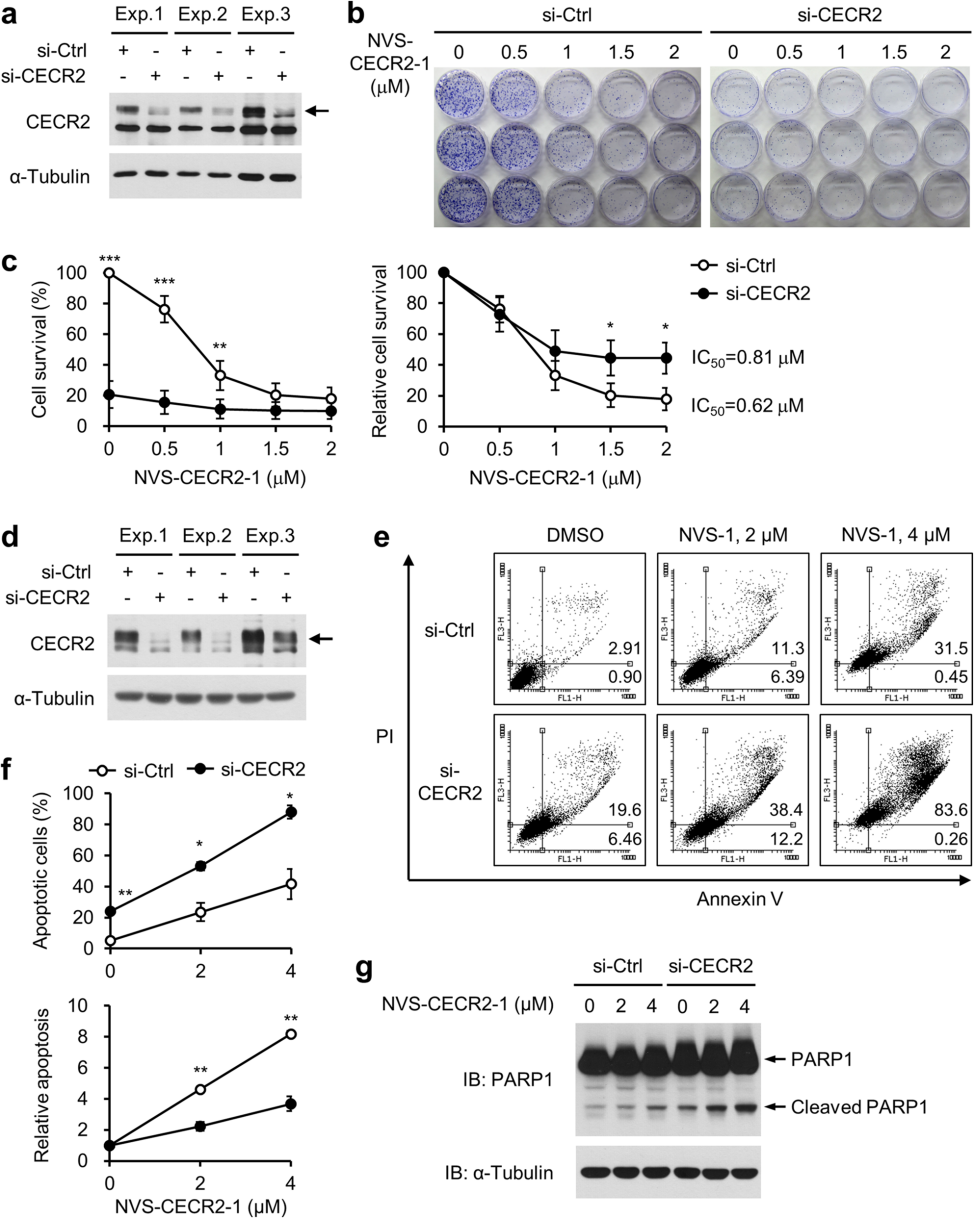
CECR2 depletion reduces the sensitivity of SW48 cells to NVS-CECR2-1. SW48 cells were transfected with control or CECR2-specific siRNAs for 48 h, and treated with increasing concentrations of NVS-CECR2-1 before being subjected to colony formation, annexin V/PI staining and PARP1 cleavage assays. (**a**) Immunoblot analysis of CECR2 knockdown of three independent experiments for colony formation assay. Arrow, CECR2 band. (**b**) Representative results of colony formation assay. (**c**) Relative cell survival in the colony formation assay was depicted as a graph by setting the value of DMSO-treated (0 μM) si-control cells as 100 (left) or by setting each of the DMSO-treated si-control and si-CECR2 cells as 100 (right). *n* = 3 (each performed in triplicate); error bars, mean ± s.d. *, *p* < 0.05; **, *p* < 0.01; ***, *p* < 0.001. (**d**) Immunoblot analysis of CECR2 knockdown of three independent experiments for apoptosis assay. Arrow, CECR2 band. (**e**) Representative results of the apoptosis assay by annexin V/PI staining (72 h after NVS-CECR2-1 treatment). (**f**) Relative apoptotic cells were depicted as a graph by percentages or by setting each of the DMSO-treated si-control and si-CECR2 cells as 1 (bottom). *n* = 3 (each performed in triplicate); error bars, mean ± s.d. *, *p* < 0.05; **, *p* < 0.01. (**g**) Results of the PARP1 cleavage assay (36 h after NVS-CECR2-1 treatment). A representative of three experiments showing similar results was shown.

Next, we asked whether NVS-CECR2-1 kills SW48 cells by targeting CECR2. If NVS-CECR2-1 exerts its cytotoxic activity by inhibiting CECR2, depletion of CECR2 should affect the cells’ sensitivity to NVS-CECR2-1. SW48 cells were depleted for CECR2 by specific siRNA (Fig. 5a) and treated with increasing NVS-CECR2-1 from 0.5 to 2 µM. The clonogenic ability of the cells were then determined by colony formation assay. NVS-CECR2-1 showed a dose-dependent cytotoxic activity on the cells transfected with nonspecific control siRNA with the IC_50_ value of 0.62 μM. Depletion of CECR2 by specific siRNA largely decreased the cell viability, indicating that CECR2 was essential for the survival of SW48 cells. Notably, NVS-CECR2-1 exhibited very little effect on the CECR2-depleted cells compared to the control cells, likely due to the large decrease of their viability (Fig. 5b,c), indicating that the sensitivity of the cells to NVS-CECR2-1 was reduced by CECR2 depletion. Nonetheless, NVS-CECR2-1 treatment still decreased the viability of the CECR2-depleted cells in a dose-dependent manner and this decreasing effect was slightly but significantly less than that on the control cells, accompanied with a shift of the IC_50_ value from 0.62 to 0.81 µM (Fig. 5b,c). We confirmed these findings by analyzing apoptosis by Annexin-V assay. NVS-CECR2-1 increased apoptosis in a dose-dependent manner as observed before. CECR2 depletion largely increased apoptosis and the rate NVS-CECR2-1 increased apoptosis of the CECR2-depleted cells was lower than that of the control cells (Fig. 5d–f). Consistently, whereas NVS-CECR2-1 increased PARP1 cleavage in a dose-dependent manner, CECR2 depletion resulted in a large increase of PARP1 cleavage, which was not further enhanced by NVS-CECR2-1 (Fig. 5g). These results showed that NVS-CECR2-1 exerts the cytotoxic activity on SW48 cells by targeting CECR2 although it still has this activity to some extent even after CECR2 depletion.

### CECR2-independent cytotoxic activity of NVS-CECR2-1

**Figure 6:**
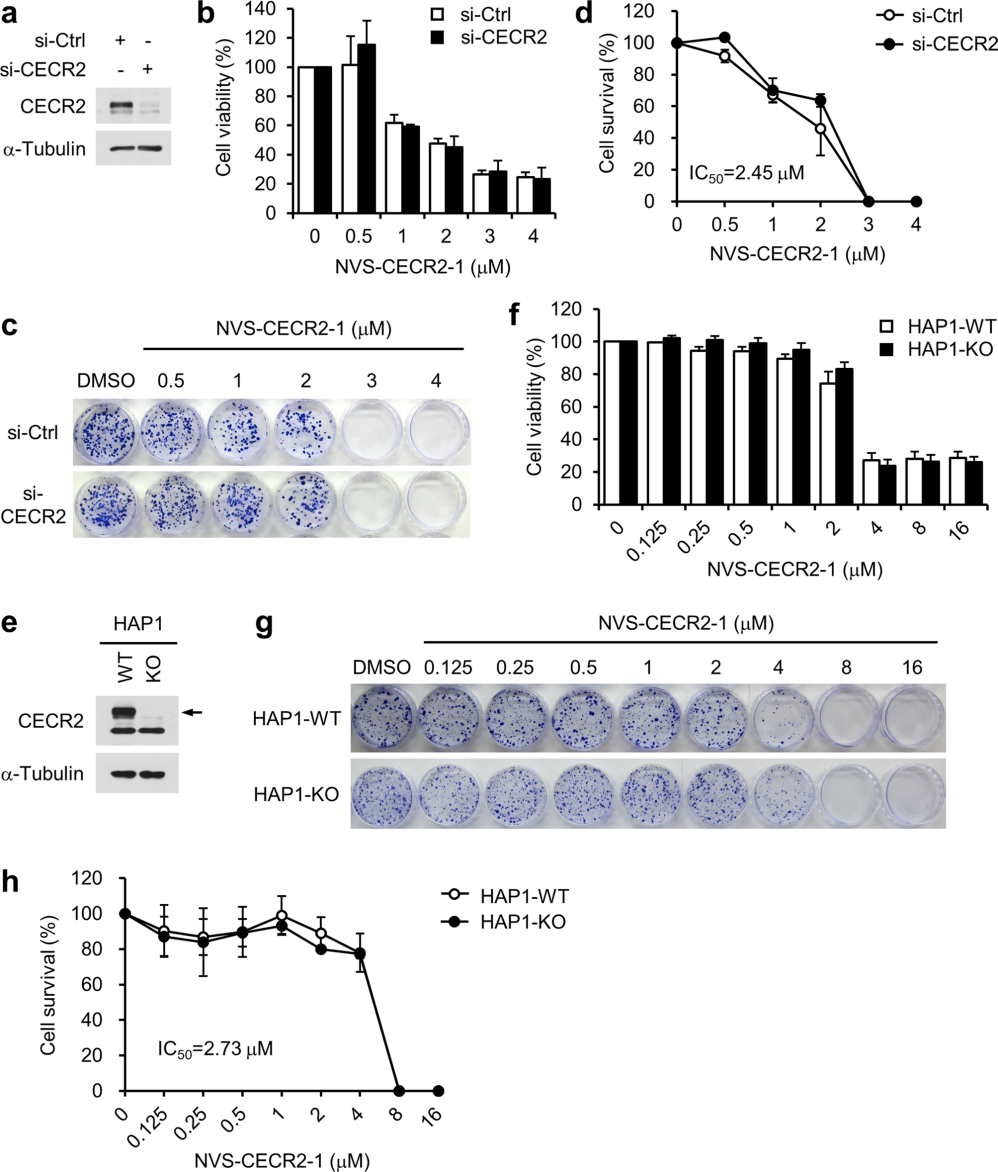
NVS-CECR2-1 exhibits cytotoxicity on 293 T and HAP1 cells independently of CECR2. (**a-d**) 293 T cells were transfected with control or CECR2-specific siRNAs for 48 h (**a**), and treated with increasing concentrations of NVS-CECR2-1 for 72 h or 12 days before being subjected to MTS (**b**) and colony formation assays (**c**, **d**), respectively. *n* = 3 (each performed in triplicate); error bars, mean ± s.d. The IC_50_ value was shown and virtually same between si-control and si-CECR2 cells. (**e–h**) CECR2-wild type and—knockout HAP1 cells (**e**) were treated with increasing concentrations of NVS-CECR2-1 for 72 h or 12 days before being subjected to MTS (**f**) and colony formation assays (**g**, **h**), respectively. *n* = 3 (each performed in triplicate); error bars, mean ± s.d. The IC_50_ value was shown and virtually same between the HAP1-WT and HAP1-KO cells. Arrow, CECR2 band.

The reason that CECR2-depleted cells were still sensitive to NVS-CECR2-1 could be due to incomplete CECR2 knockdown or an off-target activity of NVS-CECR2-1. To address this issue, we determine the effect of NVS-CECR2-1 depletion on HEK 293 T cells, which were shown to be independent of CECR2 for their viability^18^. As expected, CECR2 depletion had no effect on the viability of HEK 293 T cells (Fig. 6a, b–d). Notably, NVS-CECR2-1 exhibited a dose-dependent cytotoxic activity on the control and CECR2-depleted cells to a similar degree in both short-term (Fig. 6b) and long-term viability assays (Fig. 6c,d), indicating that NVS-CECR2-1 exerted the cytotoxic activity independently of CECR2. To further substantiate these results, we analyzed the HAP1 cells, a near-haploid human cell line derived from the chronic myelogenous leukemia cell line KBM-7, in which the gene coding for CECR2 was knocked out by the CRISP/Cas9 system. Absence of CECR2 was confirmed for this cell (Fig. 6E). The establishment of this cell line itself indicates that CECR2 is dispensable for cell survival and proliferation. Indeed, we observed no difference in cell growth between the CECR2-knockout HAP1 and the parental CECR2-wild type HAP1 cells (data not shown). NVS-CECR2-1 showed a similar degree of cytotoxic activity on the both cell lines in short-term (Fig. 6f) and long-term viability assays (Fig. 6g,h). The IC_50_ values of NVS-CECR2-1 for 293 T and HAP1 cells were 2.45 and 2.73 µM, respectively, which were approximately four times higher than that for SW48 cells. Taking thus far results together, we concluded that NVS-CECR2-1 exerts cytotoxic activity not only by targeting CECR2 but also via CECR2-independent mechanism, possibly the off-target effects.

### Cytotoxic activity of NVS-CECR2-1 on various human colon cancer cells

**Figure 7:**
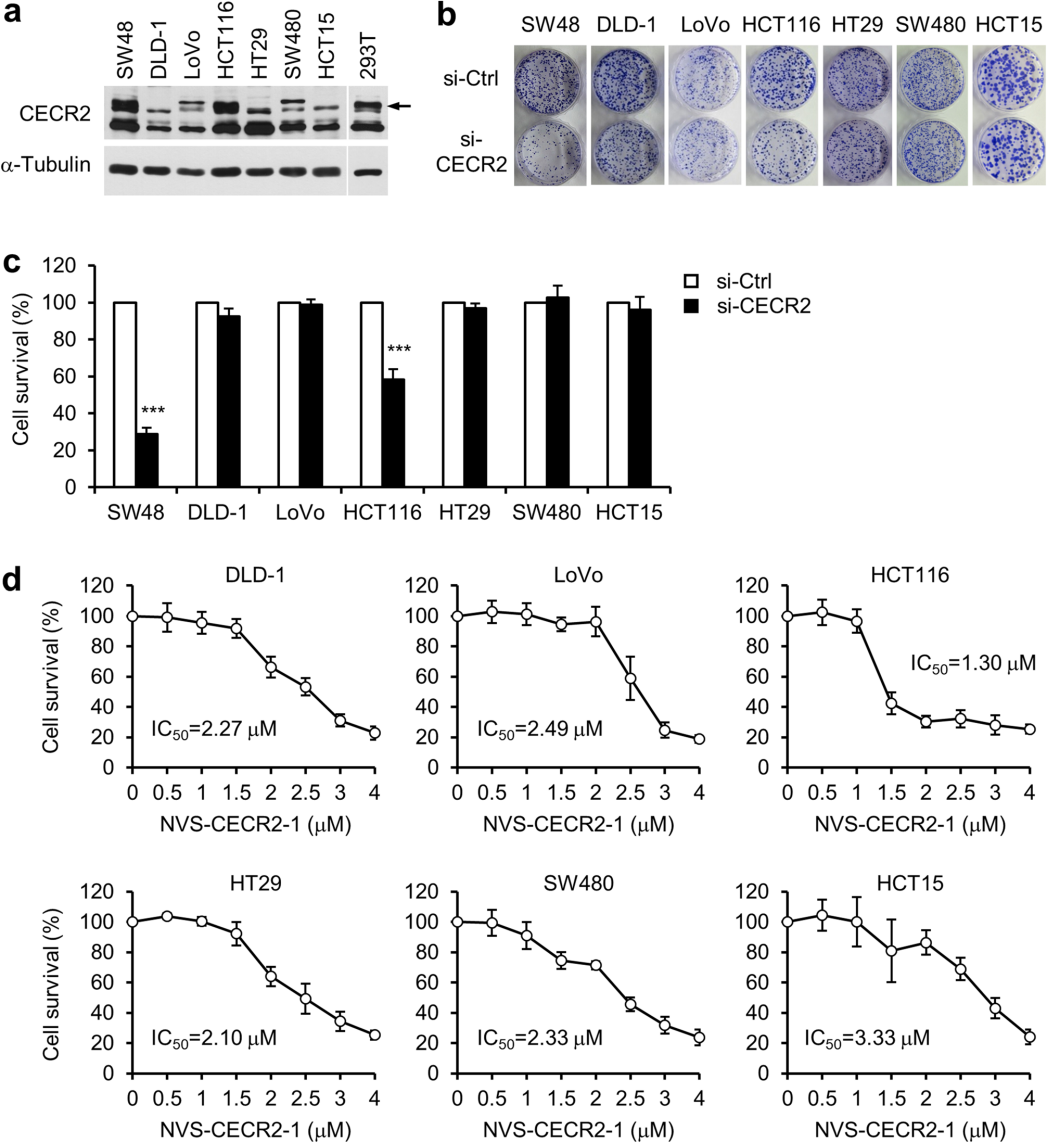
Cytotoxic activity of NVS-CECR2-1 on various human colon cancer cells. (**a**) A panel of human colon cancer cell lines were analyzed for CECR2 expression by immunoblotting. The blot of 293 T cells was taken from a separate gel with same exposure for comparison. Arrow, CECR2 band. (**b**) The cells were transfected with control or CECR2-specific siRNAs for 48 h, and the viability was determined by colony formation assay after 10 days. Representative images were shown. (**c**) Relative cell survival in the colony formation assay in (**b**) was depicted as a graph by setting the value of si-control as 100 for each cell line. n = 3 (each performed in triplicate); error bars, mean ± s.d. ***, *p* < 0.001. (**d**) The cells were treated with increasing concentrations of NVS-CECR2-1 and the viability was determined by colony formation assay after 10 days. n = 3 (each performed in triplicate); error bars, mean ± s.d. The IC_50_ value for each cell line was shown.

The results thus far suggested that cells dependent on CECR2 for their viability tend to be more sensitive to NVS-CECR2-1 than CECR2-independent cells. To determine whether this tendency can be generalized, we analyzed a set of human colon cancer cell lines, including DLD-1, LoVo, HCT116, HT29, SW480 and HCT15 as well as SW48 cells. Immunoblot analysis revealed that SW48 and HCT116 cells expressed CECR2 at the similar levels as HEK 293 T cells whereas the remaining cell lines exhibited very low levels of CECR2 expression (Fig. 7a). CECR2 depletion reduced the viability of SW48 and HCT116 cells by approximately 72% and 42%, respectively, and had no effect on the viability of the other five cell lines (Fig. 7b,c). Then, we determined the IC_50_ values of NVS-CECR2-1 against these cancer cells as described before. Notably, HCT116 cells exhibited 1.30 µM of IC_50_ for NVS-CECR2-1, which was higher than SW48 cells (0.64 µM), whereas the IC_50_ values against the other five CECR2-independent cell lines ranged from 2.1 to 3.3 µM (Fig. 7d). These results showed a good correlation between the cytotoxic activity of NVS-CECR2-1 on the cancer cells and the extent to which their survival relies on CECR2.

### Analysis of CECR2 gene expression in colorectal cancer tissues

**Figure 8:**
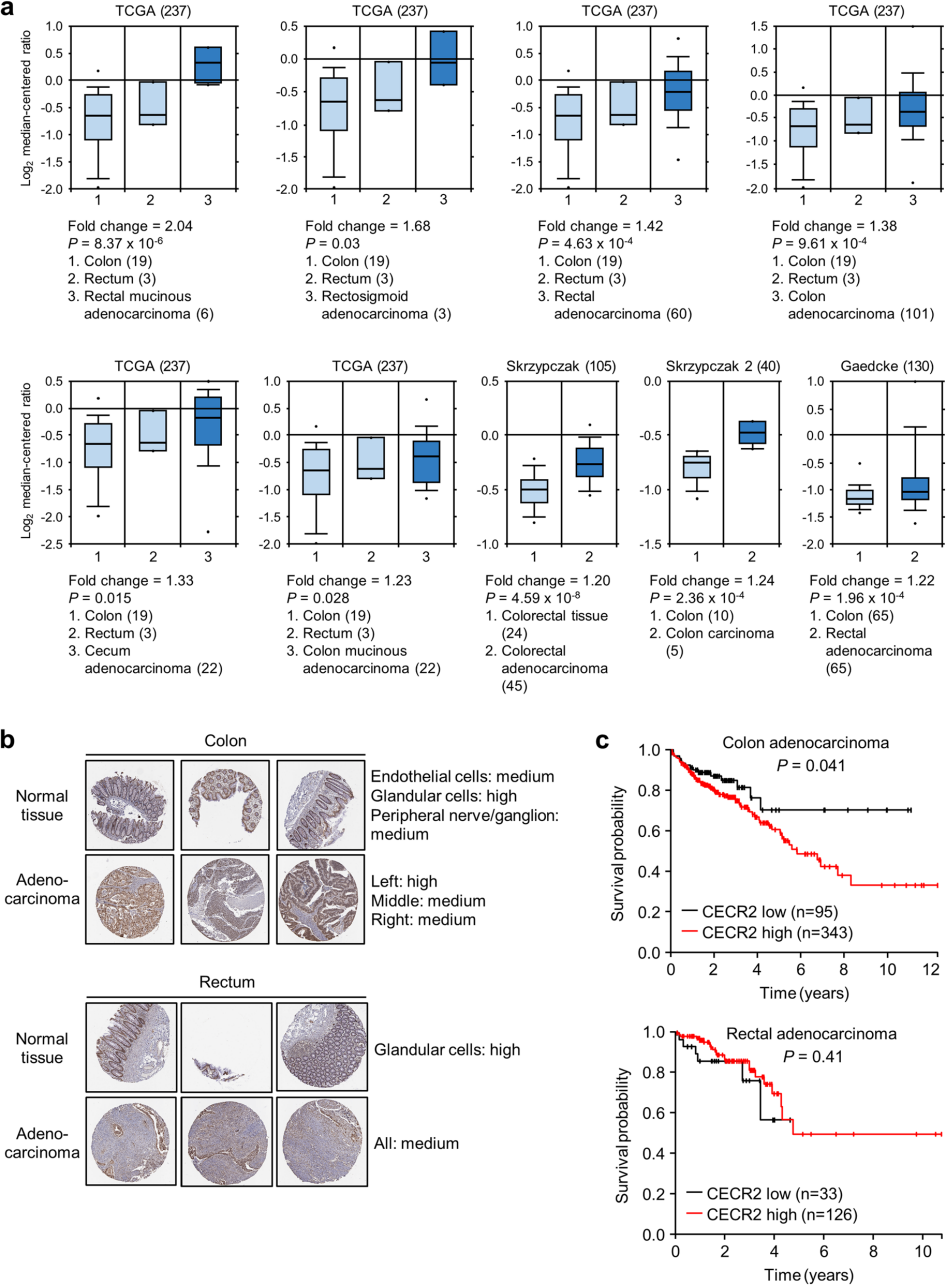
Analysis of CECR2 gene expression in human colorectal cancer. (**a**) Results of a differential analysis of colorectal normal and colorectal cancer tissues for CECR2 mRNA expression using the Oncomine database (https://www.oncomine.org/resource). Only output results satisfying ≥ 1.2 fold change and *p* < 0.05 were taken. Figure in the parentheses indicates sample number. (**b**) CECR2 protein expression was determined in colon/rectum normal and adenocarcinoma tissues by immunohistochemistry. Antibody staining in the annotated cell types in the current human tissue is reported as not detected, low, medium, or high. This score is based on the staining intensity and fraction of stained cells. The images were taken from the database of the Human Protein Atlas (https://www.proteinatlas.org/). (**c**) Kaplan–Meier plots showing the correlation between CECR2 mRNA expression level and patient survival in colon and rectal adenocarcinomas. The graphs were adapted from the database of the Human Protein Atlas.

To evaluate the clinical significance of our findings, we analyzed the CECR2 gene expression in human colorectal cancer using several online databases. First, the data from TCGA Pan-Cancer Atlas showed that CECR2 mRNA expression in colorectal cancer ranked at a moderate to medium level among the 32 different cancer types, comprising 10,967 samples from 10,953 patients (Fig. S3). A differential analysis of normal and cancer tissues in the 11 colorectal datasets comprising 2033 samples showed that CECR2 mRNA expression was upregulated in colorectal cancer tissues compared to colorectal normal tissues (Fig. 8a). Immunohistochemical staining data from the Human Protein Atlas revealed that colorectal cancer belonged to a group in which 100% of examined patients displayed medium or high levels of CECR2 protein expression (Fig. S4). However, both colorectal normal and colorectal adenocarcinoma tissues showed medium or high levels of CECR2 staining (Fig. 8b). A Kaplan–Meier survival curve indicated that the group of colon adenocarcinoma patients with high CECR2 expression showed significantly worse survival than that with low CECR2 expression (*P* = 0.041), but CECR2 was not categorized as a prognostic gene in colon cancer (gene with *P* < 0.001 is defined as prognostic) (Fig. 8c). There was no significant correlation between patient survival probability and CECR2 expression in rectal adenocarcinoma (Fig. 8c). Thus, it is not conclusive at the moment whether CECR2 is a therapeutic target for colorectal cancer and NVS-CECR2-1 is clinically applicable in treatment of this cancer type.

## Discussion

In the present study, we investigated the recently developed BRD inhibitor NVS-CECR2-1 for its potential cytotoxic activity on human cancer cells. First, we demonstrated that NVS-CECR2-1 inhibits chromatin binding of CECR2 BRD and dissociates CECR2 from chromatin within cells. Then, we found that NVS-CECR2-1 has cytotoxic activity against various human cancer cells with different tissue origins at pharmacologically significant concentrations. In particular, NVS-CECR2-1 kills SW48 colon cancer cells with a submicromolar IC_50_ value and this activity is exerted mainly by increasing apoptosis. The cytotoxic activity of NVS-CECR2-1 against the cancer cells is reduced by CECR2 depletion and is positively correlated with the extent to which the cells rely on CECR2 for their survival, suggesting that NVS-CECR2-1 exerts its activity by targeting CECR2. This is the first study that reports an anticancer cytotoxic activity for NVS-CECR2-1.

NVS-CECR2-1 was originally developed as an inhibitor specific for CECR2 BRD. It has no cross reactivity in a BRD panel of 48 targets, with only weak interactions with BRD4 and BRD7, as demonstrated by a thermal shift assay, (https://www.thesgc.org/chemical-probes/NVS-1). However, our results showed that siRNA knockdown of CECR2 in 293 T cells as previously reported^18^ or CRISPR/cas9-derived inactivation of the CECR2 gene in haploid leukemia cells did not affect the cytotoxic activity of NVS-CECR2-1 on these cells. Given the fact that CECR2 is dispensable for survival and proliferation of these two cell types, these results suggest that this activity of NVS-CECR2-1 could be exerted by off-targeting some other proteins, which are essential for cell survival and proliferation. Thus, our results have left the question open whether NVS-CECR2-1 is specific for CECR2 within cells.

CECR2 is predominantly expressed in the neural system and involved in neurulation and inner ear development during mouse embryogenesis^15,21^. CECR2 forms a complex with the ISWI family ATP-dependent chromatin remodeler SNF2L and functions in transcriptional regulation of genes involved in embryonic and post-natal brain development^15,22^. CECR2 also forms a complex with SNF2H, another ISWI family chromatin remodeler, in the testis and contributes to spermatogenesis in adult tissue^23^. The CECR2-SNF2L complex, named CERF, has been shown to remodel chromatin in vitro and displace the ATP hydrolysis activity that is stimulated by nucleosomes^15^. A genome-wide screen for human BRD-containing proteins identified CECR2 to be involved in DNA damage response^18^. However, the exact cellular functions of CECR2 remain largely unknown. Our results show that CECR2 is important for cell viability. Interestingly, the role of CECR2 in cell viability depends on cell types such that it is important for SW48 and HCT116 cells but dispensable for 293 T and KBM-7 cells. In addition, CECR2 expression levels vary even among different cancer cells with same tissue origin- a panel of colon cancer cell lines, and the requirement of CECR2 for cell viability is correlated with its expression levels. Therefore, the role of CECR2 in cell viability appears to depend on both cell type and CECR2 level. The exact role of CECR2 in the cancer cell viability and its effects on the cell sensitivity to NVS-CECR2-1 remain to be clearly defined in future study.

Motivated by the success in developing potent and highly selective inhibitors for the BET family BRDs and their entering into clinical trials in cancer treatment, research activities have been extended to developing non-BET family BRD inhibitors, including NVS-CECR2-1^13,14^. CECR2 has been ranked top among the 24 human BRD-containing proteins for the score of druggability^24^ and our work showed that NVS-CECR2-1 has < 10 μM of the IC_50_ values in cytotoxicity against various human cancer cells. Therefore, it is possible that NVS-CECR2-1 can be developed as a candidate of potential cancer therapeutic agent. In particular, our studies using a panel of human colon cancer cell lines raised the possibility that NVS-CECR2-1 has a potential to be used as a therapeutic agent in colon cancer treatment. In support of this, a differential analysis showed that CECR2 mRNA expression is upregulated in colorectal cancer tissues compared to normal counterparts. However, immunohistochemical staining of CECR2 scored high or medium levels in both colorectal normal and adenocarcinoma tissues from several patients, and CECR2 was not defined as a prognostic gene in colorectal cancer based on the Kaplan–Meier survival curves. Thus, the answer to the question whether NVS-CECR2-1 has a potential of clinical application for treatment of colon cancer is indecisive for now and reserved for future study. Given our data demonstrating the potent activity of NVS-CECR2-1 to displace CECR2 from chromatin within cells, this BRD inhibitor can also be used as a useful tool for studying the cellular functions of CECR2.

## Methods

### Cells and antibodies

HCT15, SW48, SW480, DLD-1, LoVo, H460, SV-HUC-1 and HCT116 cells were purchased from ATCC (USA), and HT29, U2OS, HEK 293 T and HeLa cells from Korean Cell line Bank (KCLB, Seoul). HCT15, SW48, SW480, DLD-1, LoVo, H460, and SV-HUC-1 cells were maintained in RPMI1640 medium supplemented with 10% fetal bovine serum (FBS), 100 U/ml penicillin, and 100 µg/ml streptomycin. HCT116, HT29, and U2OS cells were cultured in McCOY's 5A modified medium supplemented with 10% FBS, 100 U/ml penicillin, and 100 µg/ml streptomycin. HEK 293 T and HeLa cells were maintained in Dulbecco's modified Eagle's medium (DMEM) supplemented with 5% FBS, 100 U/ml penicillin, and 100 µg/ml streptomycin. HAP1 parental (C631) and CECR2-KO (HZGHC000946c001) cell lines were purchased from Horizon Discovery (Cambridge, UK) and cultured in Iscove's modified Dulbecco's medium (IMDM) supplemented with 10% FBS, 100 U/ml penicillin and 100 µg/ml streptomycin. All cells were maintained at 37 °C in a 5% CO_2_ humidified incubator. The sources of antibody used in this work are as follows; anti-CECR2 (LifeSpan Biosciences, LS-C496852), anti-BRG1 (Santa Cruz Biotechnology, sc-17796), anti-c-Myc (BIOMOL, SA-294), anti-α-Tubulin (Abcam, ab18251), anti-GAPDH (Abfrontier, LF-PA0212), anti-Lamin A/C (Cell Signaling Technology, 2032), anti-PARP1 (Santa Cruz Biotechnology, sc-8007).

### BRD inhibitors and doxorubicin

NVS-CECR2-1 was obtained from the SGC and PFI-3 was purchased from Tocris (5072, UK). Doxorubicin was purchased from Sigma. The inhibitors and drugs were dissolved in DMSO, kept in aliquots at -20 °C, and thawed immediately before use for experiments to minimize unexpected chemical reactions.

### Plasmids, siRNA and transfection

siRNA transfection was performed using Lipofectamine RNAiMAX (Invitrogen). The sequences of CECR2-specific siRNA: 5′-cugcuaucaacgaagagau(dTdT)-3′. Plasmid DNA transfection was performed using Polyethylenimine (PEI). The expression vectors for GFP-Myc-CECR2-BRD(2x) and GFP-Myc-BRG1-BRD(2x) were described previously^18,20^.

### Chromatin fractionation assay

Biochemical fractionation of chromatin binding proteins was performed as previously described^25,26^. Cells were suspended in the fraction III buffer (50 mM HEPES (pH 7.5), 150 mM NaCl, 1 mM EDTA, 0.5% NP-40, 0.5 mM PMSF, 5 µg/ml aprotinin, 5 µg/ml leupeptin, 5 µg/ml pepstatin A, and 10 mM NaF) on ice for 60 min. The lysate was subjected to centrifugation at 16000 g for 20 min to separate supernatant (soluble, cytoplasmic proteins) from pellet (insoluble, chromatin-bound proteins). We note that the fraction III buffer described above extracts chromatin-unbound or loosely bound proteins, thus separating them from chromatin-bound proteins.

The salt gradient chromatin fractionation was performed essentially in the same way as previously described^27^. Cells were lysed in CEBN buffer (10 mM HEPES (pH 7.8), 10 mM KCl, 1.5 mM MgCl_2_, 0.34 M sucrose, 10% glycerol, 0.2% NP-40, protease inhibitors as described above) on ice for 5 min, and the lysate was centrifuged to separate supernatant (cytoplasmic fraction) from pellet. After wash with CEB buffer (CEBN buffer without NP-40), the pellet was suspended in soluble nuclear buffer (3 mM EDTA, 0.2 mM EGTA, protease inhibitors as described above), incubated on ice for 5 min and centrifuged to separate supernatant (soluble nuclear fraction) from pellet. The resulting pellet was then subjected to gradient salt extraction with salt buffers with increasing NaCl concentration (50 mM Tris (pH 8.0), 0.05% NP-40, NaCl as indicated, protease inhibitors as described above).

### Immunoblot analysis

Immunoblot analysis was performed by standard method. Cells were washed with PBS and lysed in RIPA buffer (50 mM Tris–Cl (pH 8.0), 150 mM NaCl, 0.5% sodium deoxycholate, 0.1% SDS, 1% NP-40, 1 mM DTT, 0.5 mM PMSF, 5 µg/ml aprotinin, 5 µg/ml leupeptin, 5 µg/ml pepstatin A, and 10 mM NaF) on ice for 30 min. The protein concentrations were quantified by BCA method. After separation on SDS gel, proteins were transferred onto nitrocellulose membrane (GE Healthcare Life Sciences) using normal transfer buffer (25 mM Tris-base, 192 mM Glycine, and 20% methanol). Signals were detected by enhanced chemiluminescence (ECL, Sigma).

### Apoptosis assay

Annexin V/PI double staining was performed using the FITC Annexin V Apoptosis Detection Kit I (556,547, BD Bioscience) according to the manufacturer’s protocol. 6 × 10^5^ cells were washed twice with PBS and resuspended in 1-ml binding buffer. 100 µl of the suspension (1 × 10^5^ cells) were mixed with 5 µl of Annexin V-FITC and 5 µl of propodium iodide (PI, Invitrogen). After gentle vortex, cells were incubated at room temperature for 20 min in the dark before being subjected to flow cytometric analysis using a FACSCalibur (BD Biosciences).

### MTS assay

2- 4 × 10^3^ cells were seeded in 96-well plate in 100 µl of medium per well. After 72 h, MTS assays were performed according to the protocols of Cell Tilter 96 aqueous One Solution Cell Proliferation assay (Promega). The absorbance at 490 nm was measured using a spectraMax i3X plate reader (Molecular Devices, I3X-SC-ACAD).

### Colony formation assay

Colony formation assay was performed as previously described^20^. After staining with 0.5% crystal violet, colonies were dissolved in 10% acetic acid before measuring absorbance at 590 nm with a spectraMax i3X plate reader (I3X-SC-ACAD, Molecular Devices).

### Statistical analysis

The significance of differences between groups was evaluated by Student's t-test in Microsoft Excel. A *p* value less than 0.05 was considered significant. IC_50_ value was calculated by GraphPad Prism software.

## Supplementary information

Supplementary file1
