# Supplementary material for: Cytotoxic activity of bromodomain inhibitor NVS-CECR2-1 on human cancer cells
Source: Sci Rep. 2020 Oct 1;10:16330. doi: 10.1038/s41598-020-73500-7 (PMC7529788; doi:10.1038/s41598-020-73500-7)

Supplementary Information

Cytotoxic activity of bromodomain inhibitor NVS-CECR2-1 on human cancer cells

Seul Gi Park, Daye Lee, Hye-Ran Seo, Shin-Ai Lee, and Jongbum Kwon

Supplementary Figure S1

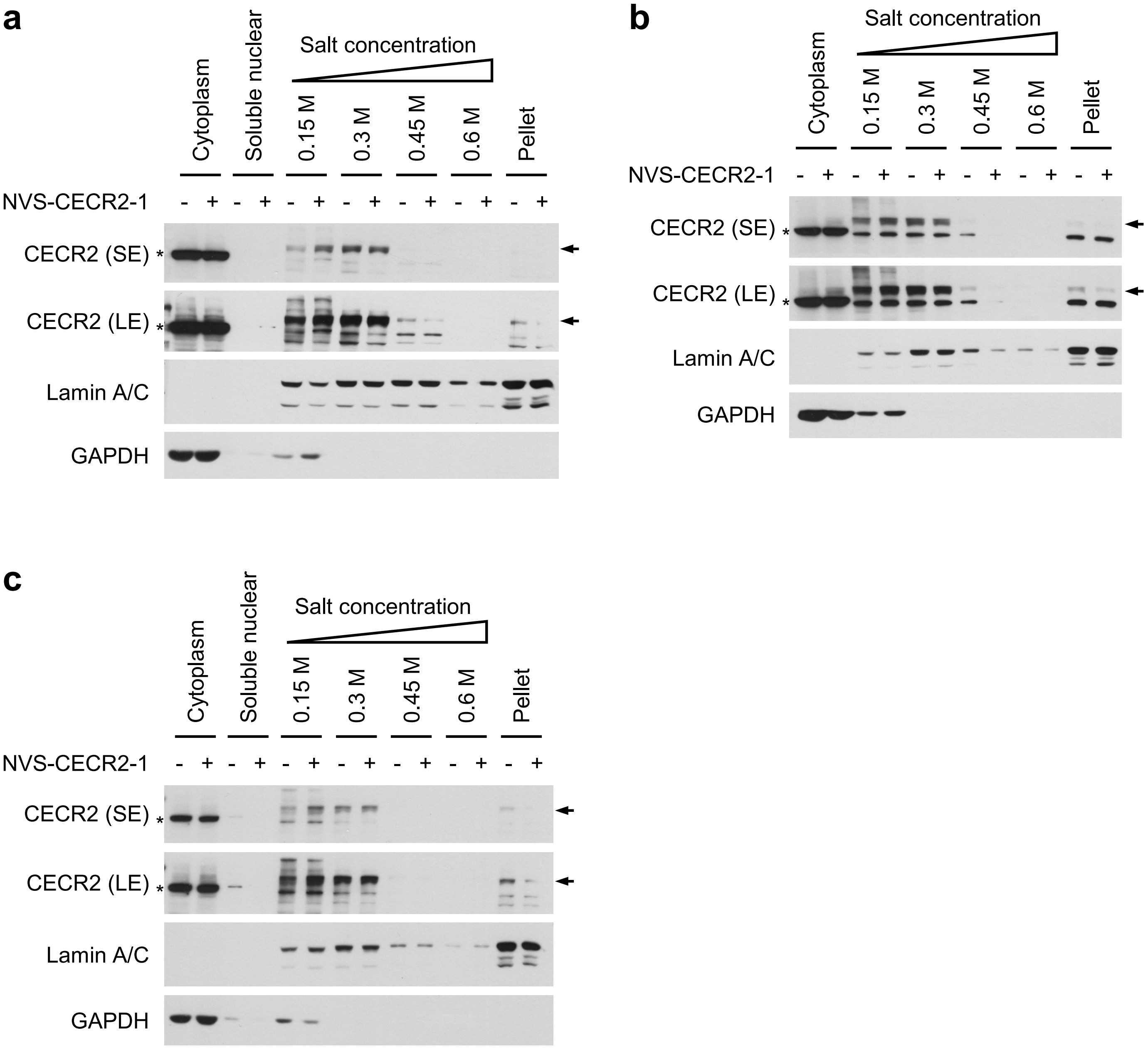

Figure S1. Results of the similar experiments as described in Fig. 2c. Arrow, CECR2 band; asterisk, nonspecific band.

Supplementary Figure S2

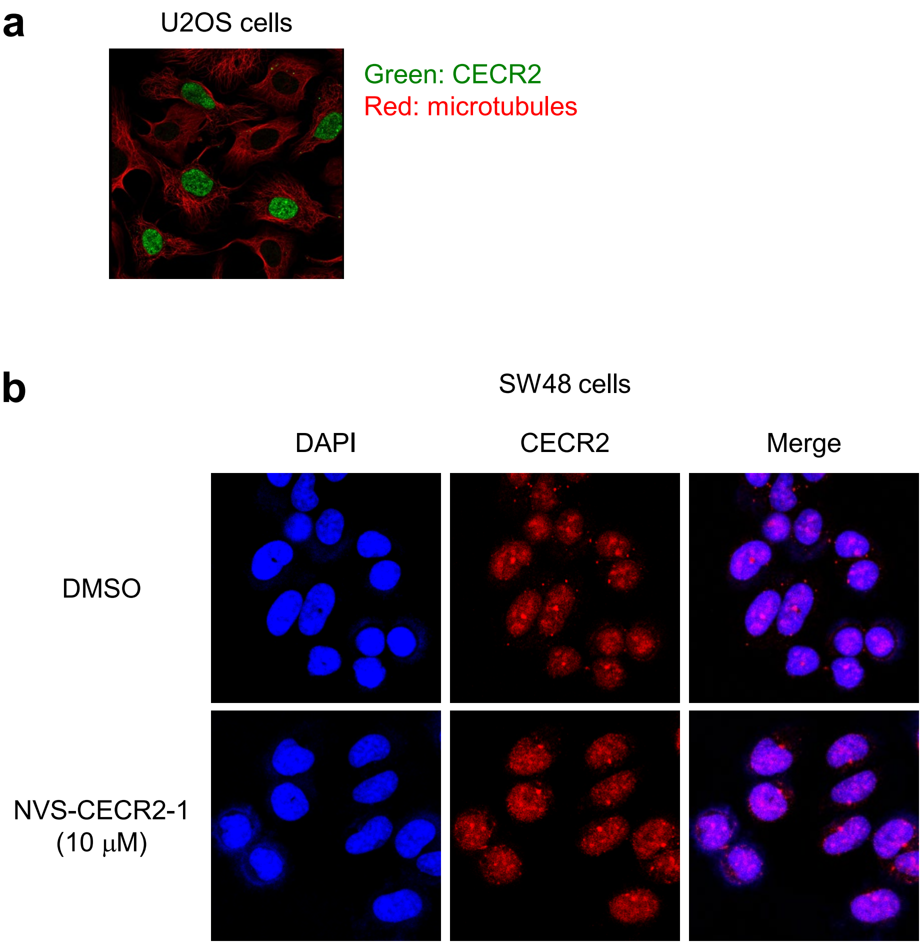

Figure S2. Immunofluorescence microscopy for CECR2. **(a)** U2OS cells were stained for CECR2 and microtubules. The images were taken from the Human Protein Atlas database (https://www.proteinatlas.org/). **(b)** SW48 cells were treated with DMSO (vehicle) or NVS-CECR2-1 at 10 μM for 2 h, fixed with formaldehyde for 10 min and permeabilized with 0.2% Triton X-100/PBS for 12 min. After blocking, the cells were incubated with anti-CECR2 antibody overnight at 4°C. After washing with PBS, the cells were incubated with secondary antibody for 30 min in the dark. The cells were washed again and mounted in Vectashield mounting medium with DAPI (H-1200, Vector Laboratories) before confocal images were captured using Carl Zeiss LSM 510.

Supplementary Figure S3

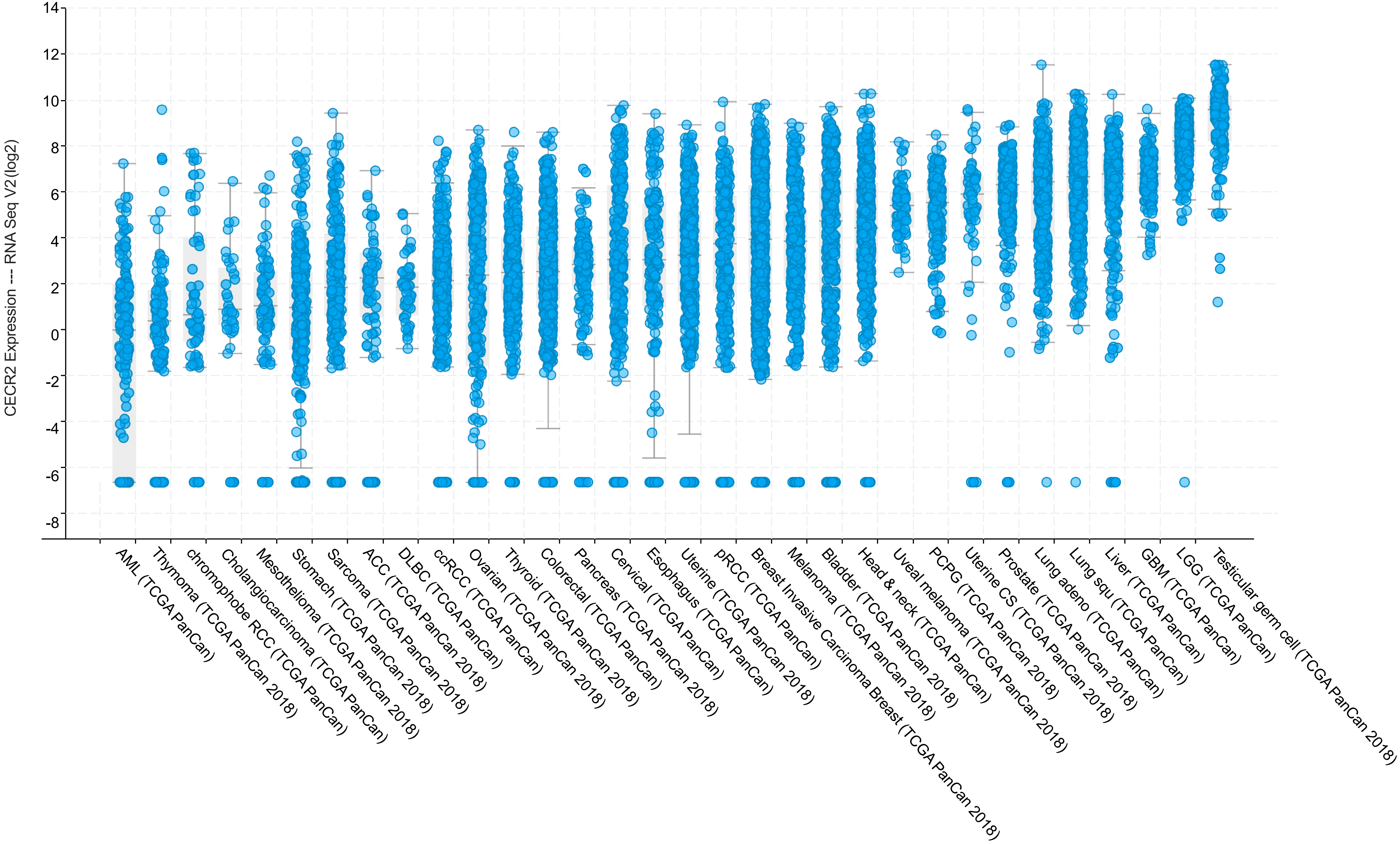

Figure S3. Result of the analysis of CECR2 mRNA expression in the studies of TCGA Pan-Can Atlas (32 cancer types). The analysis was performed using the online database of the cBioPortal for Cancer Genomics (https://www.cbioportal.org/).

Supplementary Figure S4

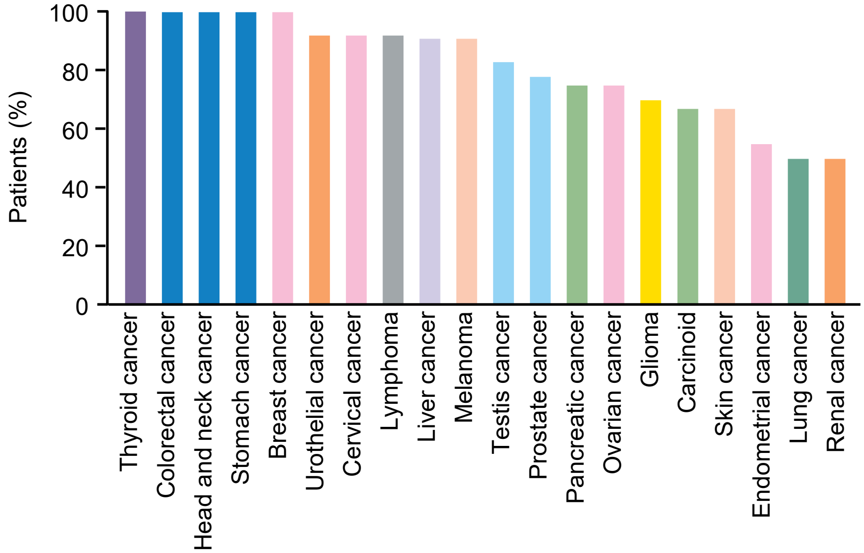

Figure S4. The CECR2 expression was determined in various caner types by immunohistochemistry. For each cancer, color-coded bars indicate the percentage of patients (maximum 12 patients) with high and medium protein expression level. For colorectal cancer, 11 of 11 patients show high/medium levels of CECR2 expression. The graph was taken from the Human Protein Atlas database (https://www.proteinatlas.org/).

Uncropped gels

Fig. 1b

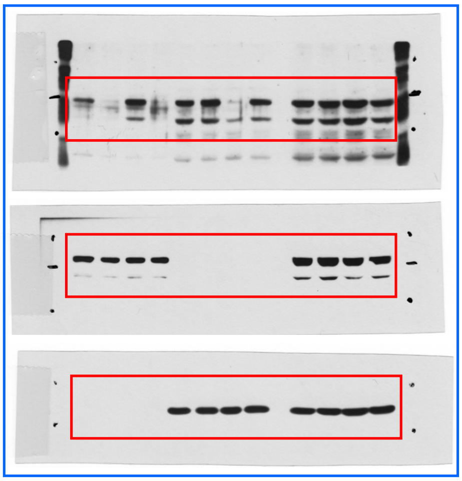

Fig. 1c

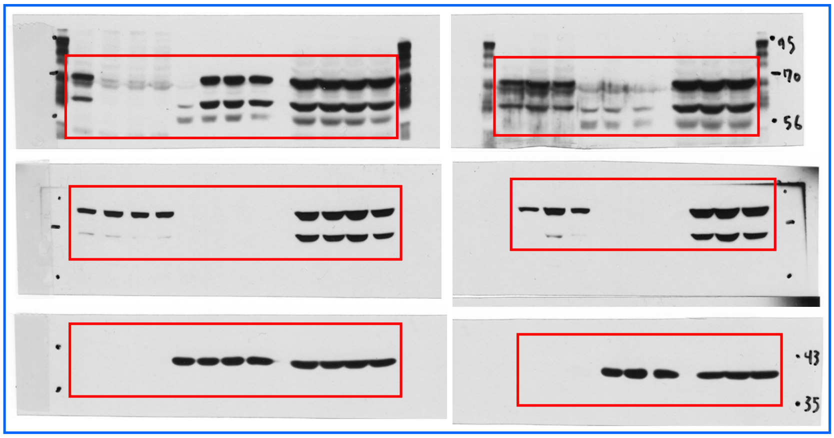

Fig. 1d

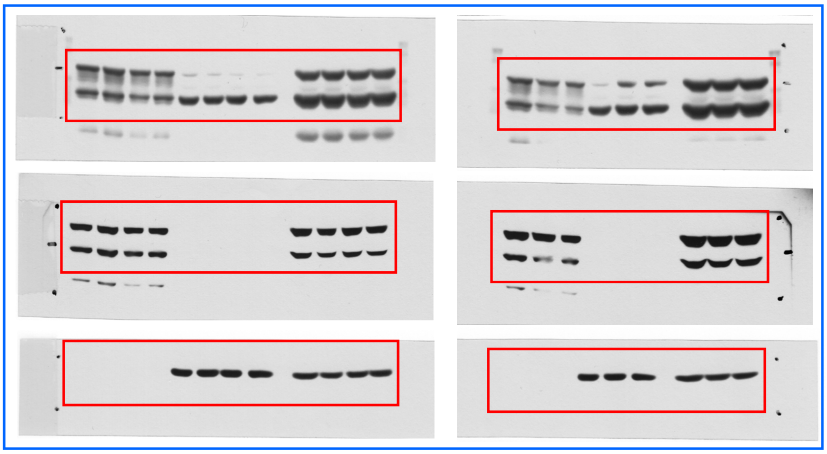

Fig. 2a

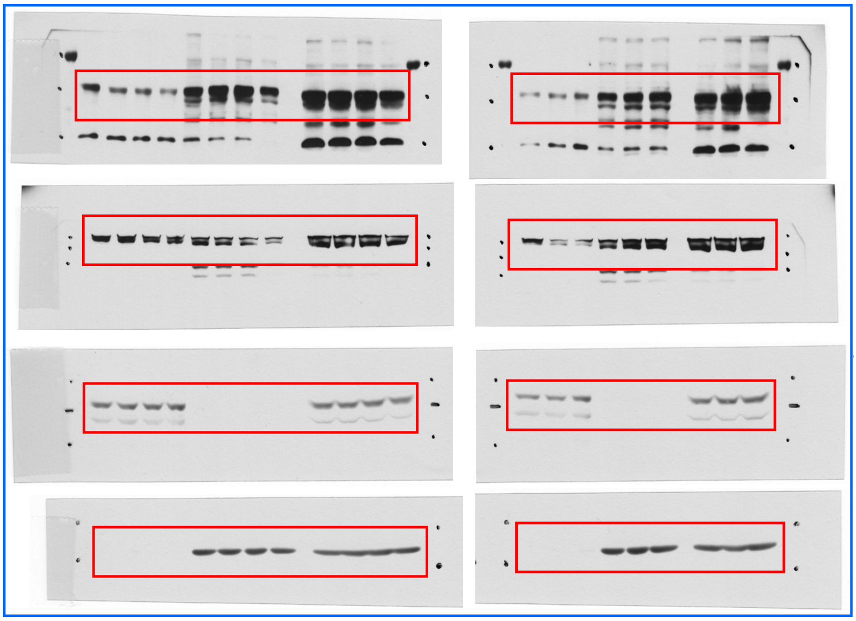

Fig. 2c

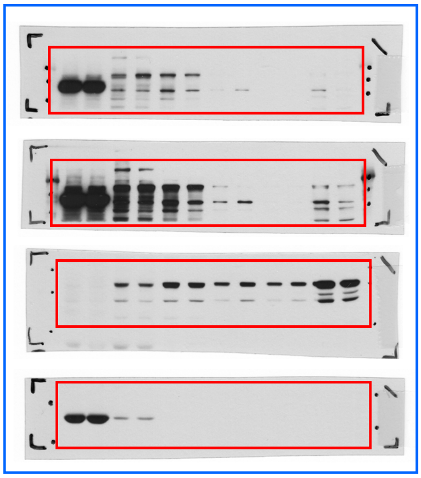

Fig. 3

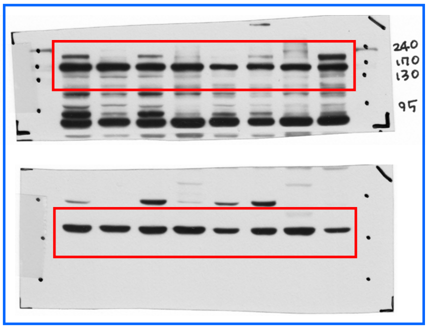

Fig. 4e

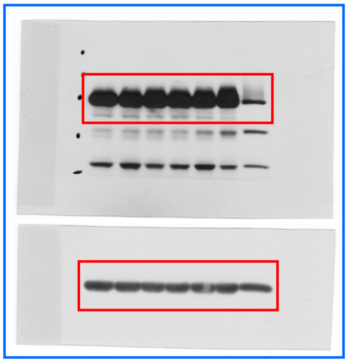

Fig. 5a

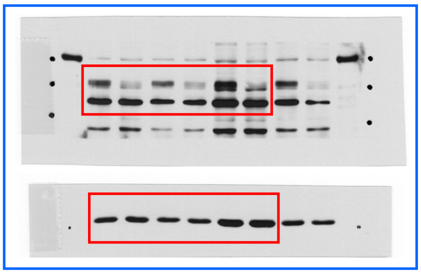

Fig. 5d

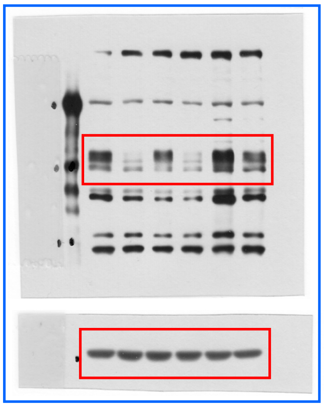

Fig. 5g

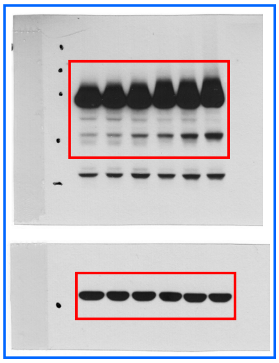

Fig. 6a

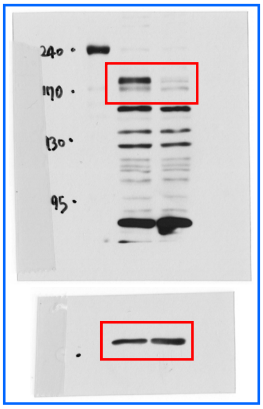

Fig. 6e

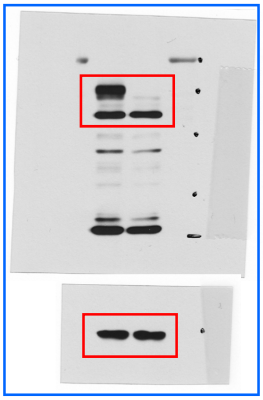

Fig. 7a

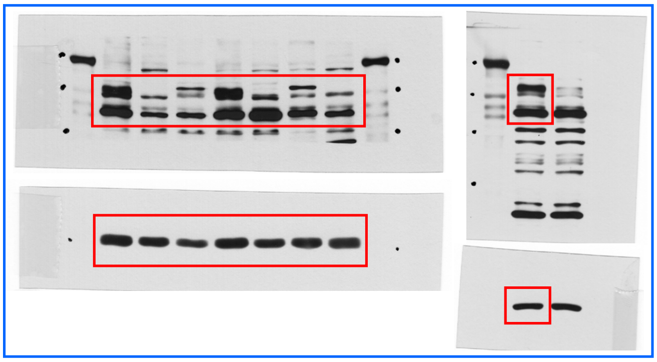

Fig. S1a

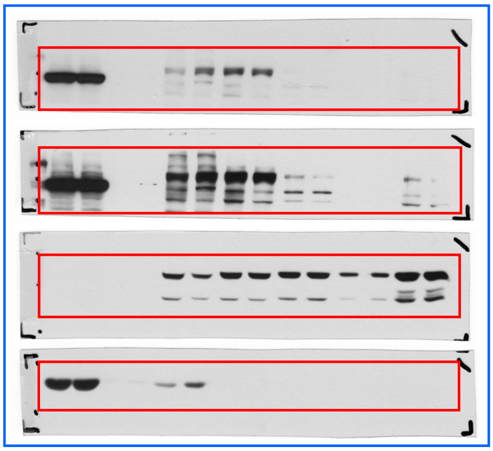

Fig. S1b

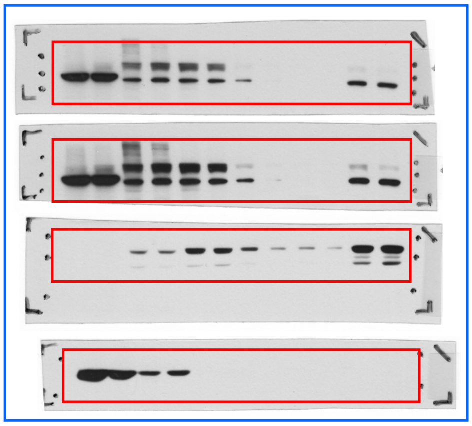

Fig. S1c

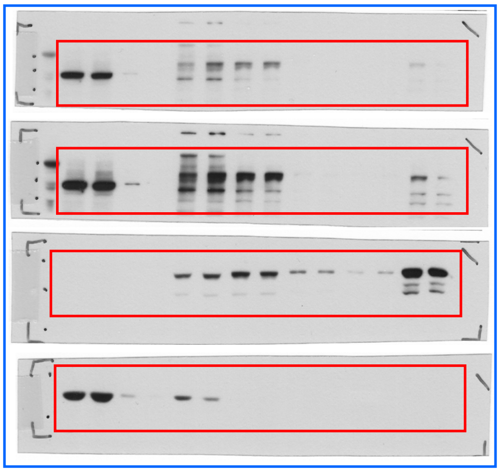

Supplement: Supplementary file 1 — Supplementary file1 [file 41598_2020_73500_MOESM1_ESM.pdf]
